# Glutathione-S-transferase M1 regulation of diesel exhaust particle-induced pro-inflammatory mediator expression in normal human bronchial epithelial cells

**DOI:** 10.1186/1743-8977-9-31

**Published:** 2012-08-06

**Authors:** Weidong Wu, David B Peden, Rob McConnell, Scott Fruin, David Diaz-Sanchez

**Affiliations:** 1 Center for Environmental Medicine, Asthma, and Lung Biology, University of North Carolina, Chapel Hill, NC, 27599, USA; 2 Department of Preventive Medicine, University of Southern California, Los Angeles, CA, 90033, USA; 3 Environmental Public Health Division, National Health and Environmental Effects Research Laboratory, US Environmental Protection Agency, Chapel Hill, NC, 27599, USA

**Keywords:** Diesel exhaust particles, ROS, GSTM1, ERK, Akt

## Abstract

**Background:**

Diesel exhaust particles (DEP) contribute substantially to ambient particulate matter (PM) air pollution in urban areas. Inhalation of PM has been associated with increased incidence of lung disease in susceptible populations. We have demonstrated that the *glutathione S-transferase M1 (GSTM1)* null genotype could aggravate DEP-induced airway inflammation in human subjects. Given the critical role airway epithelial cells play in the pathogenesis of airway inflammation, we established the *GSTM1* deficiency condition in primary bronchial epithelial cells from human volunteers with *GSTM1* sufficient genotype (*GSTM1*+) using *GSTM1* shRNA to determine whether *GSTM1* deficiency could exaggerate DEP-induced expression of interleukin-8 (IL-8) and IL-1β proteins. Furthermore, the mechanisms underlying GSTM1 regulation of DEP-induced IL-8 and IL-1β expression were also investigated.

**Methods:**

IL-8 and IL-1β protein levels were measured using enzyme-linked immunosorbent assay. *GSTM1* deficiency in primary human bronchial epithelial cells was achieved using lentiviral *GSTM1* shRNA particles and verified using real-time polymerase chain reaction and immunoblotting. Intracellular reactive oxygen species (ROS) production was evaluated using flow cytometry. Phosphorylation of protein kinases was detected using immunoblotting.

**Results:**

Exposure of primary human bronchial epithelial cells (*GSTM1*+) to 25-100 μg/ml DEP for 24 h significantly increased IL-8 and IL-1β protein expression. Knockdown of *GSTM1* in these cells further elevated DEP-induced IL-8 and IL-1β expression, implying that *GSTM1* deficiency aggravated DEP-induced pro-inflammatory response. DEP stimulation induced the phosphorylation of extracellular signal-regulated kinase (ERK) and Akt, the downstream kinase of phosphoinositide 3-kinase (PI3K), in *GSTM1*+ bronchial epithelial cells. Pharmacological inhibition of ERK kinase and PI3K activity blocked DEP-induced IL-8 and IL-1β expression. DEP-induced ERK and Akt phosphorylation could be increased by *GSTM1* knockdown. In addition, pretreatment of HBEC with the antioxidant N-acetyl cysteine significantly inhibited DEP-induced ERK and Akt phosphorylation, and subsequent IL-8 and IL-1β expression.

**Conclusion:**

*GSTM1* regulates DEP-induced IL-8 and IL-1β expression in primary human bronchial epithelial cells by modulation of ROS, ERK and Akt signaling.

## Background

Diesel exhaust particles (DEP) emitted during the combustion of diesel fuel are an important contributor to the levels of particulate matter (PM) air pollution in urban areas. These particles comprise a carbonaceous core to which organic and inorganic compounds, such as polycyclic aromatic hydrocarbons (PAHs), nitro and oxygenated derivatives of PAHs, heterocyclic compounds, aldehydes, aliphatic hydrocarbons, and heavy metals, can be adsorbed. Epidemiological and experimental studies have shown that DEP inhalation is associated with elevated incidence of diverse respiratory disorders including pulmonary inflammation, increased susceptibility to respiratory infections, increased risk of lung cancer, and exacerbation of asthma and chronic obstructive pulmonary diseases [1-4]. However, the mechanisms underlying DEP-induced pulmonary disorders have not yet been adequately elucidated.

The pathogenesis of many respiratory diseases is characterized by airway inflammation, which is driven by a plethora of pro-inflammatory mediators released from airway resident and infiltrating inflammatory cells [5]. The airway epithelium represents the interface between the external environment and the tissue of the airway wall [6]. The production of pro-inflammatory mediators from airway epithelium plays a critical role in the pathogenesis of pulmonary diseases [5,7]. Exposure to air pollution particles has been shown to evoke pro-inflammatory mediator production in airway epithelial cells [8-10]. It has been demonstrated that the pro-inflammatory effect of air particles is affected by many factors, such as particle size, concentration, composition, duration of exposure, and co-pollutants [11]. Increasing evidence indicates that the host susceptibility factors may also play an important role in air pollutant-induced lung inflammation [12,13]. Susceptibility to the adverse effects of air pollutants is an intrinsic trait most probably related to genotypes [14]. Animal studies have shown that prolonged low-dose DEP exposure induces airway inflammatory responses that differ remarkably among mouse strains with different genetic backgrounds of oxidative stress response [15]. It has been proposed that host responses to DEP are regulated by a balance between antioxidant defenses and pro-inflammatory responses [16]. The lung has multiple anti-oxidative defense systems including the glutathione *S*-transferases (GSTs) [17]. The GSTs are a supergene family of phase II conjugating enzymes that consist of a number of sub-classes such as GSTM1 and GSTP1, and catalyze the conjugation of reduced glutathione with hydrophobic electrophiles and reactive oxygen species (ROS) [18]. *GSTM1* is mapped to the *GST mu* 1 gene cluster on chromosome 1p13.3. Genetic variants that regulate the availability and functionality of the GST enzymes determine the levels of oxidative effects in the airway and associated injury [19]. *GST* gene polymorphisms, particularly the *GSTM1* null genotype, are frequent in the population with reported frequencies from 18 to 66% in different ethnic groups [20]. The deletion variants or null alleles that exist for the *GSTM1* gene present biochemically as a failure to express protein [21,22]. Individuals with the *GSTM1* null genotype completely lack the GSTM1 enzyme activity and their susceptibility to asthma and lower lung function is increased [23-25].

Our previous studies have demonstrated that the *GSTM1* null genotype is associated with aggravation of airway inflammation in human subjects exposed to diverse air toxicants including ozone, endotoxin, DEP, and second hand smoke [14,26-28], implying that *GSTM1* deficiency might be a risk factor in air pollutant-induced lung diseases. It should be noted that these in vivo studies investigated only the association of *GSTM1* genotype with pollutant-induced lung inflammation, and they cannot exclude the contribution of other genetic factors in the modulation of response to air pollutants. To our knowledge, no mechanistic studies have been conducted to examine the function of GSTM1 protein in the pathogenesis of airway inflammation. Given the critical role airway epithelial cells play in the pathogenesis of airway inflammation, we manipulated *GSTM1* levels in primary human bronchial epithelial cells (HBEC) from human volunteers with *GSTM1* sufficient (*GSTM1*+) genotype using *GSTM1* shRNA to determine whether *GSTM1* deficiency could modulate DEP-induced pro-inflammatory response, herein, the over-expression of interleukin-8 (IL-8) and IL-1β proteins. In addition, the mechanisms whereby GSTM1 regulated DEP-induced IL-8 and IL-1β protein expression were also examined.

## Results and discussion

### DEP exposure increases IL-8 and IL-1β protein expression in *GSTM1*+ primary human bronchial epithelial cells

**Figure 1:**
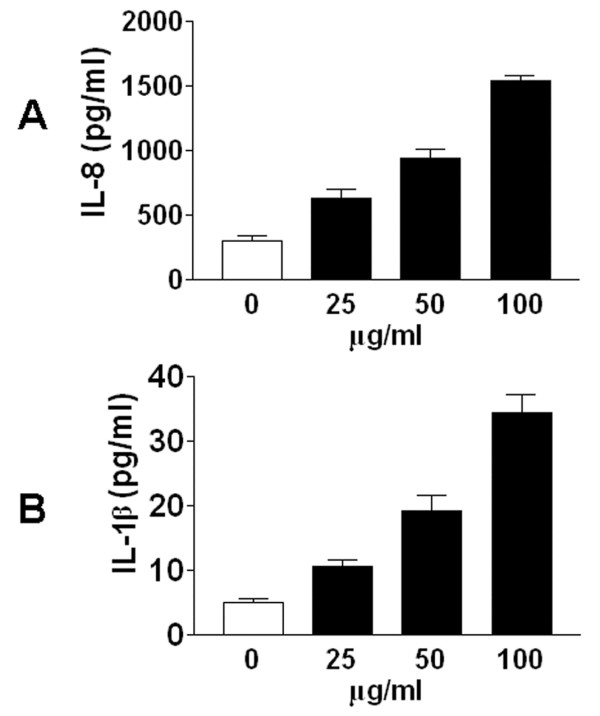
** DEP exposure increases IL-8 and IL-1β protein expression in HBEC.** Confluent HBEC were exposed to control (PBS) or 25–100 μg/ml DEP for 24 h, respectively. IL-8 and IL-1β levels in the supernatants of culture media were measured using ELISA.

IL-8 is a major mediator of acute pulmonary inflammation as a chemoattractant for neutrophils [29,30]. IL-1β is also an important mediator of the inflammatory response that can also induce production of other pro-inflammatory cytokines and chemokines [31]. Increased levels of IL-8 and IL-1β have been observed in inflammatory lung diseases [32,33]. In this study we used IL-8 and IL-1β as the biomarker of pro-inflammatory response of airway epithelial cells to DEP stimulation. Exposure of HBEC to 100 μg/ml DEP for up to 24 h did not result in significant alterations in cell viability, as assessed by assay of lactate dehydrogenase activity released into the culture medium. As shown in Figure 1A, exposure of HBEC to 25-100 μg/ml DEP for 24 h induced a significant increase in IL-8 protein expression (F = 92.36, p < 0.01). Similarly, DEP stimulation also induced a dose-dependent increase in IL-1β protein expression in HBEC (Figure 1B, F = 46.22, P < 0.01). These results indicate that DEP stimulation up-regulates IL-8 and IL-1β protein expression in *GSTM1*+ primary human bronchial epithelial cells.

In regard to the environmental relevance of the DEP concentration used in this study, a recent study has calculated that a plausible real-world exposure could result in an inhalational exposure of 0.9 mg of DEP in certain settings such as bus depots, garages and tunnels [34]. With an approximately 5% deposition throughout the conducting airways in a periciliary volume of 50-500 μl this amount of DEP would result in a concentration between 90 and 900 μg/ml. Therefore, the DEP doses used in this study (25-100 μg/ml) are relevant to real environmental exposure situations.

The DEP used in this study was suspended in molecular-grade water. It has been reported that these DEP contain both redox metals and redox active organic substances [35]. The metals appear to be tightly bound to particles and are not extractable into water. To define the contribution of metallic component to DEP-induced biological effect, normal human bronchial epithelial cells were pretreated with 100 μM deferoxamine for 2 h prior to 50 μg/ml DEP stimulation. It was shown that deferoxamine had little inhibitory effect on DEP-induced ROS production, ERK activation, as well as IL-8 expression (data not shown). The particles also contain electrophiles which exhibit both water and dichloromethane solubility. To determine the contribution of aqueous extract to DEP-induced IL-8 expression in HBEC, we centrifuged the DEP suspension at 13000 rpm for 30 min and determined the effect of the supernatant of DEP suspension on IL-8 expression in HBEC. It was found that there was no significant difference in IL-8 induction between DEP aqueous extract and control (data not shown). This suggested that water soluble components of DEP played a minimal role in DEP-induced pro-inflammatory response.

### *GSTM1* knockdown significantly increases DEP-induced IL-8 and IL-1β protein expression in HBEC

**Figure 2:**
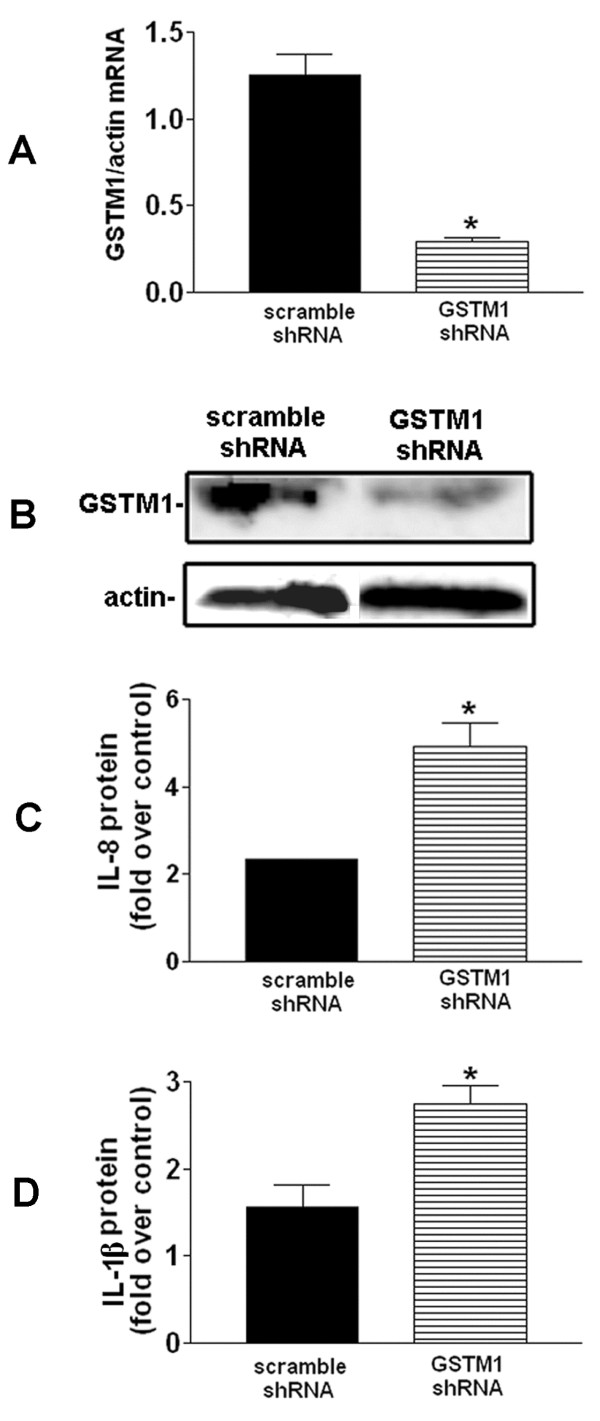
*** GSTM1*knockdown enhanced DEP-induced IL-8 and IL-1β expression in HBEC.** A and B, HBEC were infected with lentiviral scrambled or *GSTM1* shRNA particles (10 moi) for 24 h, respectively. The cells were lysed for *GSTM1* mRNA measurement using RT-PCR (*P < 0.01)or GSTM1 protein determination using immunoblotting. Infected HBEC with lentiviral scrambled or *GSTM1* shRNA particles were treated with PBS control or 50 μg/ml DEP for 24 h. Levels of IL-8 (C) and IL-1β (D) proteins in the supernatant of culture media were determined using ELISA and expressed as fold over control (DEP vs. PBS control in scrambled or *GSTM1* shRNA group, respectively). *P < 0.05, compared to DEP treatment in the scrambled shRNA group.

We have demonstrated that *GSTM1* null genotype is associated with aggravation of DEP-induced airway inflammation in human subjects. Given that the airway epithelium plays an important role in regulating pulmonary inflammatory responses and GSTM1 expression has been detected in human airway cells [36], we assumed that modulation of *GSTM1* expression levels in airway epithelial cells might affect DEP-induced IL-8 and IL-1β expression. To test this assumption, we established the *GSTM1* deficiency condition *in vitro* in HBEC (*GSTM1*+) using lentiviral *GSTM1* shRNA particles and determined its effect on DEP-induced IL-8 and IL-1β expression. This *in vitro* approach provided the opportunity of examining the contribution of *GSTM1* deficiency to DEP-induced pro-inflammatory response. HBEC (*GSTM1*+) were infected with lentiviral scrambled or *GSTM1* shRNA particles, respectively, prior to DEP treatment. As shown in Figure 2A and B, infection of HBEC (*GSTM1*+) with 10 moi of lentiviral *GSTM1* shRNA particles caused significant reduction of *GSTM1* mRNA levels (by 77%) as well as GSTM1 protein as compared to the cells infected with lentiviral scrambled shRNA particles. Then, *GSTM1* sufficient or knockdown cells were treated with PBS control or 50 μg/ml DEP for 24 h. Levels of IL-8 and IL-1β proteins in the supernatant of culture medium were measured with ELISA and expressed as fold over control. As expected, DEP stimulation increased IL-8 expression in HBEC infected with control (scrambled) shRNA particles (Figure 2B). By comparison, DEP-induced IL-8 production was further enhanced in the cells infected with lentiviral *GSTM1* shRNA particles (Figure 2B). Similarly, knockdown of *GSTM1* also increased DEP-induced IL-1β expression (Figure 2C). Taken together, these results indicated that *GSTM1* deficiency increased DEP-induced IL-8 and IL-1β expression in HBEC, which was consistent with the *in vivo* observation that linked *GSTM1* null genotype to aggravation of DEP-induced airway inflammation.

The results that we present on the effect of shRNA-mediated knockdown of *GSTM1* on the expression of the inflammatory proteins were compared to their respective controls because inter-experiment variability in the response of the cells is substantial. There are multiple factors that contribute to this variability, starting with the fact that this study was conducted on primary cultures of human airway epithelial cells, derived from several donor subjects, over a period of many months. Genetics, age of the culture, passage numbers, state of activation of the cells, etc. are all known to contribute significantly as determinants of the magnitude of the response of these cells to stimulation.

### The ERK and PI3K/Akt signaling pathways regulate DEP-induced IL-8 and IL-1β expression in HBEC

**Figure 3:**
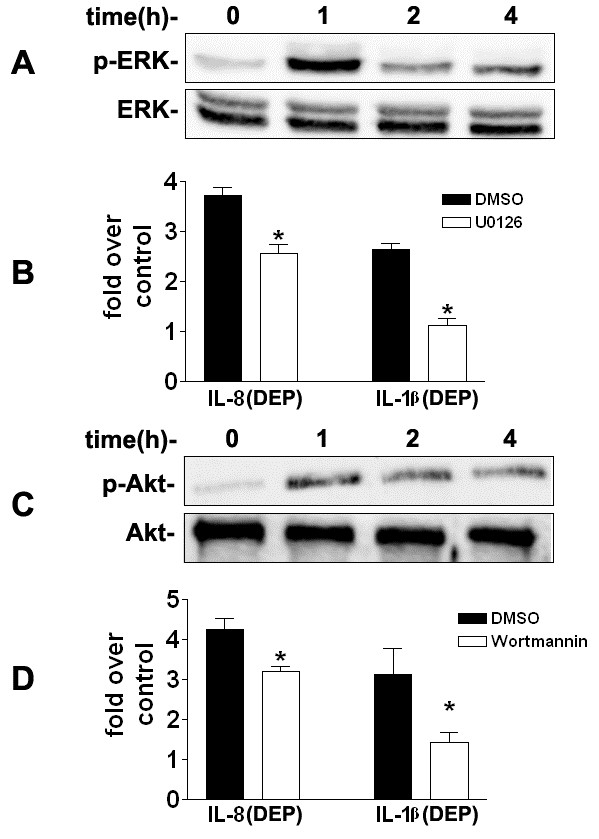
** The ERK and the PI3K/Akt pathways are involved in DEP-induced IL-8 and IL-1β expression.A**, Confluent HBEC were exposed to 50 μg/ml DEP for 1-4 h. Supernatants of cell lysates were subjected to SDS–PAGE and immunoblotting using phospho- and pan antibodies against ERK. **B**, HBEC were pretreated with vehicle (DMSO), or 20 μM ERK kinase inhibitor U0126, for 30 min prior to 50 μg/ml DEP stimulation for 24 h, respectively. Levels of IL-8 and IL-1β proteins were determined using ELISA and expressed as fold over control. *P < 0.05, compared to DEP treatment in the vehicle (DMSO) group. **C**, Confluent HBEC were exposed to 50 μg/ml DEP for 1-4 h. Phosphorylated Akt was determined using immunoblotting and phospho-specific antibody against Akt. **D**, HBEC were pretreated with vehicle (DMSO), or 1 μM PI3K inhibitor Wortmannin, for 30 min prior to 50 μg/ml DEP stimulation for 24 h, respectively. Levels of IL-8 and IL-1β proteins were measured using ELISA and expressed as fold over control (DEP vs. PBS control in vehicle DMSO or inhibitor group, respectively). * *P* < 0.05, compared to DEP treatment in the vehicle (DMSO) group.

The inflammatory responses initiated by diverse external stimulatory signals are usually regulated by activated intracellular kinases in responsive cells [37]. The rapid amplification of the initiating signal is correlated with a number of downstream protein kinases. Protein kinases have been shown to play a crucial role in the regulation of inflammatory mediator expression in the airways [38]. Previous studies have shown that the involvement of mitogen-activated protein kinases (MAPKs), including extracellular signal-regulated kinase (ERK), c-Jun NH_2_-terminal kinase (JNK), and p38 kinase pathways, and the PI3K/Akt signaling cascade, in DEP-induced up-regulation of inflammatory mediator genes is cell type-specific, and also varies greatly with pro-inflammatory mediators examined. For example, Takizawa *et al*. showed that DEPs increased intracellular adhesion molecule-1 expression through p38, but not ERK, in the transformed human bronchial epithelial cell line BEAS-2B [39]. In contrast, Boland *et al.* demonstrated that DEP stimulated granulocyte-macrophage colony-stimulating factor production mainly through ERK, and to a lesser extent, through p38 in another human bronchial epithelial cell line 16-HBE [40]. In addition, Li *et al.* found that DEP extracts could activate JNK in a human macrophage cell line THP-1[41]. In a mouse epidermal cell line DEP exposure modestly activated JNK, but had little effect on ERK and p38 [42]. In this study, we examined whether these protein kinases are involved in DEP-induced IL-8 and IL-1β expression in primary human bronchial epithelial cells. Phosphorylation of MAPKs was measured using phospho-specific antibodies against JNK, p38, and ERK, respectively. As shown in Figure 3A, exposure of HBEC to 50 μg/ml DEP induced a marked phosphorylation of ERK, but not p38 or JNK (data not shown), which peaked at 1 h of exposure. To further determine the role of ERK pathway in DEP-induced IL-8 and IL-1β production, we used the specific inhibitor of the ERK kinase U0126 to pretreat cells prior to DEP stimulation. HBEC were pre-incubated with 20 μM U0126 for 30 min prior to treatment with 50 μg/ml DEP for 24 h. IL-8 and IL-1β protein levels were measured with ELISA. As shown in Figure 3B, pretreatment of HBEC with U0126 significantly blocked DEP-induced IL-8 and IL-1β expression, indicating that the ERK signaling pathway was involved in DEP-induced IL-8 and IL-1β expression.

Next, we examined the involvement of the PI3K/Akt signaling pathway in DEP-induced IL-8 and IL-1β expression in DEP-treated HBEC. Activation of the PI3K/Akt signaling was determined by measuring the phosphorylation of Akt [43]. As demonstrated in Figure 3C, DEP stimulation (50 μg/ml) induced an acute and sustained Akt phosphorylation, indicating that the PI3K/Akt pathway was activated by DEP stimulation. To further determine whether this pathway was involved in DEP-induced IL-8 and IL-1β expression, wortmannin, the selective inhibitor of the PI3K, was used to pretreat HBEC. HBEC were pretreated with 1 μM wortmannin for 30 min before further treatment with 50 μg/ml DEP for 24 h. As shown in Figure 3D, wortmannin pretreatment inhibited DEP-induced IL-8 and IL-1β expression. These results showed that the PI3K/Akt signaling pathway is activated by DEP stimulation, further up-regulating IL-8 and IL-1β expression.

It has been proposed that the expression of inflammatory genes can be regulated at both transcriptional and posttranscriptional levels [44,45]. Exactly how the ERK and PI3K/Akt signaling pathways up-regulate DEP-induced IL-8 and IL-1β expression remains to be defined.

### Knockdown of *GSTM1* further increases DEP-induced ERK and Akt activities

The possible mechanisms underlying GSTM1-modulated lung inflammation are largely unknown. GSTM1 detoxifies electrophilic compounds by catalyzing their conjugation with reduced GSH. It is presumed that intermediate electrophilic metabolites, arising in the first phase of detoxification, are not metabolized in *GSTM1*-null asthma patients and are not excreted. These intermediate metabolites may damage cells and generate oxidative stress, and thereby contribute to the pathogenesis of asthma [46]. In addition to this well-characterized catalytic activity, recent evidence has suggested that GSTM1 may control oxidative stress and inflammation through the regulation of intracellular signaling pathways by its effects on certain small molecules or by protein-protein interactions with critical kinases. The ligand-binding capacity of GSTM1 results in the negative regulation of signaling pathways through sequestration of signaling kinases [47].

**Figure 4:**
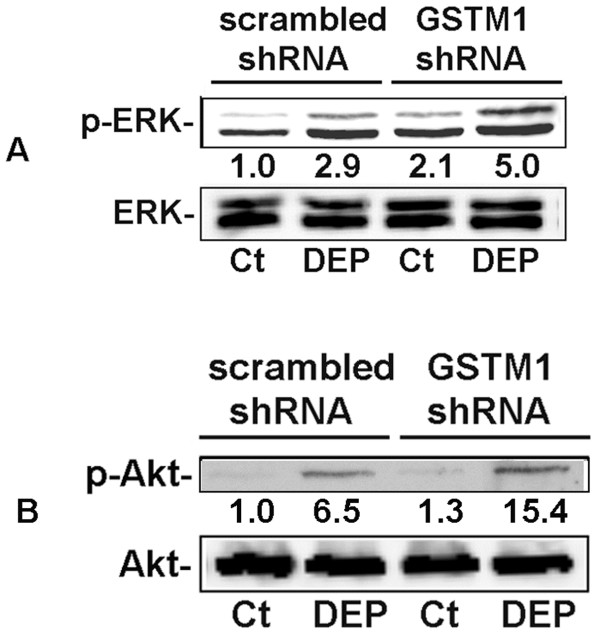
*** GSTM1*knockdown enhances DEP-induced ERK and Akt activation.** HBEC were infected with lentiviral scrambled or *GSTM1* shRNA particles (10 moi) for 24 h, respectively. Confluent cells were treated with PBS control (Ct) or 50 μg/ml DEP for 1 h. The supernatants of the cell lysates were subjected to immunoblotting using phospho- or pan antibodies against ERK (**A**) or Akt (**B**), respectively. Shown are representative bands derived from 4 separate experiments. Nos. are mean relative optical densities of phorspho-proteins compared to control.

As demonstrated previously, GSTM1, ERK and Akt were all involved in the regulation of DEP-induced IL-8 and IL-1β expression in HBEC. We hypothesized that enhancement of DEP-induced IL-8 and IL-1β protein expression by *GSTM1* deficiency might be achieved through modulation of ERK and Akt activities. To test this hypothesis, we reduced *GSTM1* expression levels in HBEC with *GSTM1* shRNA and examined ERK and Akt phosphorylation induced by DEP exposure. HBEC with sufficient or deficient *GSTM1* were treated with 50 μg/ml DEP for 1 h. Phosphorylation of ERK and Akt was determined using immunoblotting, respectively. In the cells expressing sufficient *GSTM1* DEP stimulation increased both ERK and Akt phosphorylation (Figure 4A and B). In contrast, in the cells with reduced *GSTM1* expression the phosphorylation levels of ERK and Akt induced by DEP exposure were modestly enhanced, indicating that *GSTM1* deficiency could promote DEP-induced ERK and Akt activation.

**Figure 5:**
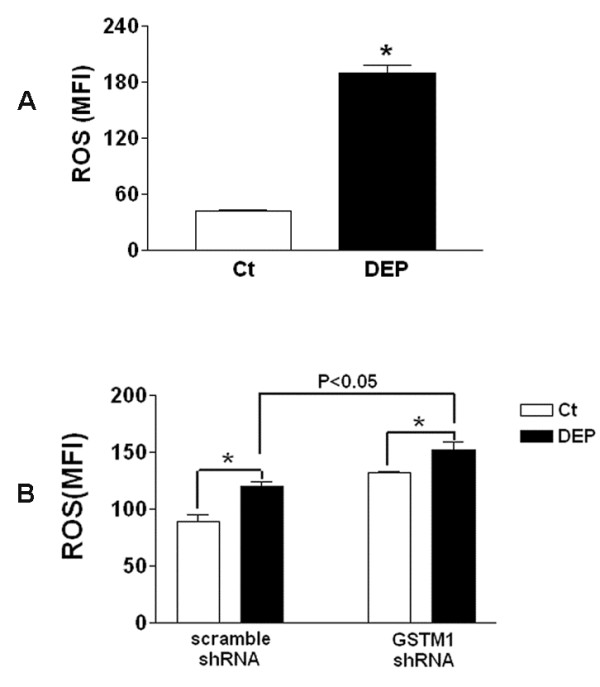
*** GSTM1*knockdown enhances DEP-induced ROS production.A**, HBEC were treated with 50 μg/ml DEP for 1 h. Intracellular ROS levels were measured using the fluorescent probe carboxy-H_2_DCFDA and flow cytometry. **B**, HBEC were infected with 10 moi of lentiviral non-target (scrambled) or *GSTM1* shRNA particles for 24 h, respectively. Confluent cells were treated with 50 μg/ml DEP for 4 h and ROS levels measured as described previously. *, compared to PBS control (Ct), *P* < 0.05.

The mechanisms whereby *GSTM1* deficiency modulated DEP-induced ERK and PI3K/Akt activation were under speculation. Given the oxidative property of many air pollutants and the feature of ROS as the second messenger in intracellular signaling network [48,49], we envisioned that the anti-oxidant GSTM1 might affect ERK and Akt activity through modulation of intracellular ROS production in HBEC exposed to DEP. The two main organic compounds adsorbed on DEP, PAHs and quinones, have been demonstrated to contribute to ROS production through enzymatic or non-enzymatic reactions [41,50-54]. DEP-induced intracellular ROS production was measured in this study. It was shown that 50 μg/ml DEP could significantly increase levels of ROS after 1 h stimulation (Figure 5A). To further examine the effect of *GSTM1* deficiency on DEP-induced ROS production, we reduced intracellular *GSTM1* levels using lentiviral *GSTM1* shRNA particles and then compared the difference in ROS production from HBEC expressing sufficient or deficient *GSTM1* after DEP treatment. *GSTM1* sufficient or deficiency cells were treated with 50 μg/ml DEP for 4 h and ROS levels measured. As shown in Figure 5B, in the cells infected with control shRNA DEP stimulation markedly increased ROS production. In contrast, in the cells containing *GSTM1* shRNA DEP-induced ROS production was further increased, indicating that *GSTM1* deficiency can increase the production of intracellular ROS in DEP-treated HBEC, possibly resulting in enhanced ERK and PI3K/Akt activation.

### Effect of the antioxidant NAC on intracellular ROS levels, ERK and Akt phosphorylation, and IL-8 and IL-1β expression

**Figure 6:**
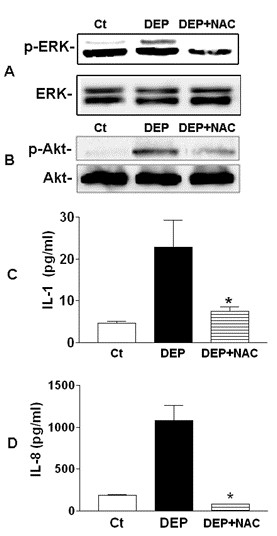
** NAC reduced DEP-induced ERK and Akt phosphorylation, and IL-8 and IL-1β expression.A** and **B**, HBEC were pretreated with 10 mM NAC for 2 h before further stimulation with 50 μg/ml DEP for 1 h. Phosphorylated ERK and Akt was measured using imunoblotting. **C** and **D**, HBEC were pretreated with 10 mM NAC for 2 h before further stimulation with 50 μg/ml DEP for 24 h. Levels of IL-8 and IL-1β proteins were measured using ELISA. *, compared to DEP, *P* < 0.05.

To further examine the involvement of ROS in DEP-induced cellular responses as described previously, we pretreated HBEC with N-acetyl-L-cysteine (NAC) prior to DEP stimulation (50 μg/ml). The antioxidant NAC is a thiol-reducing agent that can antagonize cellular ROS. Levels of phosphorylated ERK and Akt, and IL-8 and IL-1β protein were measured. As shown in Figure 6, pretreatment with NAC significantly inhibited DEP-induced ERK and Akt phosphorylation, as well as IL-8 and IL-1β expression. Taken together, these data suggested that ROS played an important role in DEP-induced ERK and Akt activation, and subsequent up-regulation of IL-8 and IL-1β. NAC is derivative of the amino acid cysteine and can be proposed to boost levels of glutathione, the substrate of GSTM1. Therefore, the fact that NAC supplementation inhibited DEP-induced oxidative and pro-inflammatory effect supported the role GSTM1 played against airway inflammation.

## Conclusion

This *in vitro* study using primary human bronchial epithelial cells provides experimental evidence in support of the notion that the *GSTM1* null phenotype is a risk factor for DEP-induced airway inflammation. Specifically, knockdown of *GSTM1* leads to enhanced IL-8 and IL-1β expression in primary human bronchial epithelial cells exposed to DEP. Furthermore, this study demonstrates that *GSTM1* knockdown increases DEP-induced IL-8 and IL-1β expression through ROS-associated ERK and Akt activation.

## Methods

### Reagents

5-(and-6)-carboxy-2´, 7´-dichlorodihydrofluorescein diacetate (carboxy-H_2_DCFDA) was purchased from Invitrogen Corporation (Carlsbad, CA). Deferoxamine (DFO) mesylate was purchased from Sigma-Aldrich (St. Louis, MO). N-acetyl-L-cysteine (NAC), U0126 and wortmannin were obtained from EMD Chemicals (Gibbstown, NJ). The rabbit antibodies against phospho-ERK and Akt, and pan ERK and Akt were obtained from Cell Signaling Technology (Beverly, MA). Horseradish peroxidase (HRP)-conjugated goat anti-rabbit antibody was obtained from Santa Cruz Biotechnology (Santa Cruz, CA). IL-8 and IL-1β ELISA assay kits were purchased from eBioscience (San Diego, CA). Chemiluminescence reagents were obtained from Thermo Scientific (Huntsville, AL). MISSION lentiviral non-target shRNA and GSTM1 shRNA transduction particles were purchased from Sigma-Aldrich Corporation (St. Louis, MO).

### Cell culture

Primary human bronchial epithelial cells (HBEC) were obtained from healthy adult human volunteers by brush biopsy of the mainstem bronchus using a cytology brush during fiberoptic bronchoscopy, conducted under a protocol approved by the Committee on the Protection of the Rights of Human Subjects at the University of North Carolina at Chapel Hill. HBEC were initially plated in supplemented bronchial epithelial cell basal medium (0.5 ng/ml human epidermal growth factor, 0.5 μg/ml hydrocortisone, 5 μg/ml insulin, 10 μg/ml transferrin, 0.5 μg/ml epinephrine, 6.5 ng/ml triiodothyronine, 50 μg/ml gentamycin, 50 ng/ml amphotericin-B, 52 μg/ml bovine pituitary extract and 0.1 ng/ml retinoic acid) on tissue culture flasks and expanded in the same growth media.

### DEP sample and preparation

The DEP used in this study was one (DEP5) of the seven DEP samples generated at the US Environmental Protection Agency’s National Risk Management Research Laboratory, Research Triangle Park, North Carolina, USA, using a 30 kW (40 hp) 4-cylinder indirect injection Deutz diesel engine (Model BF4M1008) under load of a 22.3 kW (30 hp) Saylor-Beall air compressor (Model 707). The exhaust was diluted with ambient air (3:1) to near ambient temperatures (~35°C) and directed to a small 4.2 m^3^/min (150 ft^3^/min) rated Dustex bag house (Model T6-35-9) containing Nomex felt bags. The bags were periodically reversed pulsed using compressed air to remove the accumulated DEP which were collected from the hopper at the end of each day and stored refrigerated (41°C) in glass sample jars for the *in vitro* assays [35]. The percentage of extractable organic matter (EOM) of DEP was about 31%. These particles contained high concentrations of lower-molecular-weight polycyclic aromatic hydrocarbon, phenanthrene, fluoranthene, pyrene, and metals [34].

DEP stored in the glass sample jar, as described previously, were suspended in molecular grade water (Mediatech Inc, Manassas, VA) to make a stock solution of 1 mg/ml, and sonicated just before incubated with HBEC. The particle size was less than 0.45 μm.

### Enzyme linked immunosorbent assay (ELISA)

After exposure of HBEC to DEP for 24 h, the culture media were collected and centrifuged. Levels of IL-8 and IL-1β proteins in the supernatants were measured with human IL-8 and IL-1β ELISA kits following the manufacturer's instructions.

### Immunoblotting

HBEC exposed to DEP were washed twice with ice-cold phosphate-buffered saline (PBS), and then lysed in RIPA buffer (1x PBS, 1% nonidet P-40, 0.5% sodium deoxycholate, 0.1% SDS, and protease inhibitors: 20 μg/ml leupeptin, 20 μg/ml aprotinin, 0.5 mM phenylmethylsulfonyl fluoride, 200 μM sodium orthovanadate, and 20 mM sodium fluoride). The supernatants of cell lysates were subjected to SDS-PAGE. Proteins were transferred onto nitrocellulose membrane. Membrane was blocked with 5% nonfat milk, washed briefly, incubated with primary antibody at 4°C overnight, followed by incubating with corresponding HRP-conjugated secondary antibody for 1 h at room temperature. Immunoblot images were detected using chemiluminescence reagents and the Fujifilm LAS-3000 imaging system (Fuji Medical Systems, USA).

### *GSTM1* knockdown assay

5 × 10^4^ HBEC were placed in a 12 well plate and grown overnight. 10 moi (multiplicity of infection) of lentiviral non-target (scrambled) or *GSTM1* shRNA particles in 0.5 ml bronchial epithelial growth medium were incubated with HBEC for 24 h. The infection medium was removed and replaced with fresh growth medium. Upon confluence, HBEC were lysed and assayed for *GSTM1* mRNA levels and GSTM1 protein, respectively.

### Real-time polymerase chain reaction

HBEC infected with lentiviral scrambled or *GSTM1* shRNA particles were lysed with TRIZOL reagent (Invitrogen Corporation, Carlsbad, CA) and RNA extracted. Total RNA (100 ng), 0.5 mM NTP (Pharmacia, Piscataway, NJ), 5 μM random hexaoligonucleotide primers (Pharmacia, NJ), 10 U/μl RNase inhibitor (Promega, CA), and 10 U/μl Moloney murine leukemia virus RT (GIBCO-BRL Life Technologies) were incubated in a 40°C water bath for 1 h in 50 μl of 1x PCR buffer to synthesize first-strand cDNAs. The reverse transcription was inactivated by heating at 92°C for 5 min. Oligonucleotide primer pairs and fluorescent probes for *GSTM1* and *actin* were obtained from Applied Biosystem (Carlsbad, CA).

Quantitative fluorogenic amplification of cDNA was performed using the ABI Prism 7500 Sequence Detection System (Perkin-Elmer, CA). The relative abundance of *GSTM1* mRNA levels was calculated using the difference between the cycle threshold (CT) of the *GSTM1* mRNA sequence and the reference *actin* mRNA sequence.

### Measurement of intracellular reactive oxygen species (ROS)

The intracellular formation of ROS in HBEC was detected using the fluorescent ROS probe carboxy-H_2_DCFDA. Carboxy-H_2_DCFDA is a cell-permeant indicator for ROS that is nonfluorescent until the acetate groups are removed by intracellular esterases and oxidation occurs within the cell [55]. The green fluorescence produced by HBEC is proportional to the amount of ROS produced. Briefly, confluent HBEC were pre-incubated with 20 μM carboxy-H_2_DCFDA at 37°C for 1 h prior to exposure to 50 μg/ml DEPs. Cells were detached by 0.05% trypsin-EDTA, washed once with PBS, suspended in 0.5 ml PBS and put on ice before determination of green fluorescence intensity. Flow cytometry was performed with a FACSORT (Becton–Dickinson, Miami, FL, USA) by using an argon-ion laser (wavelength 488 nm). The FACSORT was calibrated with Calibrite beads before each use, and 6000 events were counted for all sample runs. Relative cell size and density/granularity were quantified by analyzing light-scatter properties using CellQuest software (Becton–Dickinson), namely forward scatter for cell size and side scatter for density/granularity, and recording the mean fluorescence intensities for each.

## Statistical analysis

Data are presented as means±SE. Data were evaluated using nonparametric paired t tests with the overall α level set at 0.05. One-way ANOVA was used to analyze the dose-dependent trends of IL-8 and IL-1β protein expression.

## Abbreviations

DEP: Diesel exhaust particles; GST: Glutathione S-transferase; ROS: Reactive oxygen species; MAPKs: Mitogen-activated protein kinases; ERK: Extracellular signal-regulated kinase; PI3K: Phosphoinositide 3-kinase; PAHs: Polycyclic aromatic hydrocarbons; JNK: C-Jun NH_2_-terminal kinase; HBEC: Human bronchial epithelial cells; PBS: Phosphate-buffered saline.

## Competing interests

The authors declare that they have no competing interests.

## Authors’ contributions

WW, DBP, RM, and DDS designed this study. WW performed the experiments, acquisition and statistical analysis of data. The manuscript was written by WW and revised by DBP, RM, DDS, and SF. All authors read, corrected and approved the manuscript.
